# LC-MS Characterization and Biological Activities of Cuban Cultivars of *Plectranthus neochilus* Schltr

**DOI:** 10.3390/plants11010134

**Published:** 2022-01-04

**Authors:** Annarli O. Rodríguez-Ferreiro, Ania Ochoa-Pacheco, Daniel Méndez-Rodriguez, Emilia Ortiz-Beatón, Oneida Font-Salmo, Frenkel Guisado-Bourzac, Silvia Molina-Bertrán, Lianet Monzote, Paul Cos, Kenn Foubert, Luc Pieters, Claudina Perez-Novo, Wim Vanden Berghe, Julio C. Escalona-Arranz, William N. Setzer

**Affiliations:** 1 Department of Biomedical Engineering, Faculty of Telecom, Informatics and Biomedical Engineering, Universidad de Oriente, Santiago de Cuba 90500, Cuba; anmarliolivia@uo.edu.cu (A.O.R.-F.); eobeaton@uo.edu.cu (E.O.-B.); ofs@uo.edu.cu (O.F.-S.); 2 Pharmacy Department, Faculty of Natural and Exact Sciences, Universidad de Oriente, Santiago de Cuba 90500, Cuba; aochoap@uo.edu.cu (A.O.-P.); smolina@uo.edu.cu (S.M.-B.); 3 Chemistry Department, Faculty of Applied Chemistry, University of Camagüey, Camagüey 74650, Cuba; daniel.mendez92r@gmail.com; 4 Laboratory of Applied Genetic and Genomic, School of Sea Sciences, Pontificia Universidad Católica de Valparaiso, Valvaraiso 2362807, Chile; Frenkel.guisado.b@mail.pucv.cl; 5 Department of Parasitology, Institute of Tropical Medicine “Pedro Kourí”, Havana 11400, Cuba; monzote@ipk.sld.cu; 6 Research Network Natural Products against Neglected Diseases (ResNetNPND), University of Münster, 48149 Münster, Germany; paul.cos@uantwerpen.be; 7 Laboratory of Microbiology, Parasitology and Hygiene (LMPH), Faculty of Pharmaceutical, Biomedical and Veterinary Sciences, University of Antwerp, 2610 Antwerp, Belgium; 8 Natural Products & Food Research and Analysis (NatuRA), Faculty of Pharmaceutical, Biomedical and Veterinary Sciences, University of Antwerp, 2610 Antwerp, Belgium; kenn.foubert@uantwerpen.be (K.F.); luc.pieters@uantwerpen.be (L.P.); 9 Laboratory for Protein Chemistry, Proteomics and Epigenetic Signaling, Faculty of Pharmaceutical, Biomedical and Veterinary Sciences, University of Antwerp, 2610 Antwerp, Belgium; Claudina.PerezNovo@uantwerpen.be (C.P.-N.); wim.vandenberghe@uantwerpen.be (W.V.B.); 10 Aromatic Plant Research Center, 230 N 1200 E, Suite 100, Lehi, UT 84043, USA

**Keywords:** *Plectranthus neochilus*, diterpenes, antimicrobial, sedative, essential oil

## Abstract

*Plectranthus neochilus* Schltr. (Lamiaceae) is a plant recently introduced in Cuba. Worldwide, it is an ethnomedicinal alternative for its use against microbial infections, but the Cuban population use the extracts to treat sleep disorders. To address this apparent incongruity, four collections (from different seasonal conditions in the year) of Cuban *P. neochilus* cultivars were analyzed in terms of their pharmacognostic characteristics. Three extracts using fresh and dried leaves were chemically and biologically characterized. UPLC-DAD-MS/MS analysis was performed to determine their chemical composition, while a panel of nine microorganisms was used to evaluate their antimicrobial activity. Finally, cytotoxic effects of different fractions were measured in three cell lines by the resazurin viability assay. In contrast to previously reported micro and macromorphological properties of *P. neochilus*, the leaves from the Cuban cultivars did not present glandular trichomes, nor did they produce quantifiable levels of essential oils. Moreover, aqueous extracts used by the population revealed no significant antimicrobial activity and were not cytotoxic. The three extracts showed a similar phytochemical composition, i.e., eight flavonoids, seven abietane diterpenes, and rosmarinic acid as the major constituent, most of them reported for the first time in this species. The low yield of essential oil, the absence of glandular trichomes, compounds with a high level of oxidation, and a moderate antimicrobial activity detected were the most distinctive pharmacognostic and biological characteristics of *P. neochilus* grown in Cuba. These aspects could explain its non-use as an antimicrobial.

## 1. Introduction

The World Health Organization (WHO) highlights that about 80% of the population from developing countries make use of medicinal plant remedies as a cost-effective way to solve health problems. Low-income countries have limited access to expensive commercial drugs on the international market, which forces people to resolve health issues with natural herbal plants/remedies [1]. In this context, natural products or natural product derivatives continue to lead the process of obtaining new entities in drug discovery. In fact, of the 1881 new drugs approved in the period between 1981 and 2019, 787 (41.8%) are directly related to a natural origin. In addition, 489 analogs were synthesized that mimic natural compounds, which together represent two-thirds of the novel drugs [2]. The importance of natural products in health management also becomes apparent from the average growth rate of the global trade of medicinal herbal derivatives, reaching an estimated market of USD 83 billion in 2014 [3]. Therefore, natural medicines should be the focus of underdeveloped countries not only as a way to solve their own health problems but also to get access to new income sources.

All those factors increase the common practice to introduce non-native medicinal plants in countries, with the purpose to reproduce this source of raw material either by private/individual or governmental entities for medical/commercial purposes. However, this intention to cultivate non-native medicinal plants will not always lead to the exact reproduction of the bioactive properties that characterize them in their natural habitat [4]. Intrinsic factors can be better controlled by determining the identity of the plant, as well as its genetic authenticity, but the external factors, such as environment, cultivation, harvest, and post-harvest processing/storage practices, become more difficult to manage, especially when the activity is developed by the population [5].

*Plectranthus neochilus* Schltr., an aromatic and succulent species from the Lamiaceae, is one of these plants whose cultivation has spread to almost the entire world. The endemic plant from South Africa and Namibia is commonly known as ‘blue coleus’ or ‘lobster flower’ (English speakers), ‘muskietbossie’ by Africans, and ‘boldo-rasteiro’ by Portuguese. It is traditionally used in African and Brazilian folk medicine to treat skin diseases, respiratory ailments, disturbed digestion, hepatic insufficiency, and dyspepsia [6,7]. From the pharmacologic point of view, antioxidant [8], antibacterial [9], cytotoxic [10], and α-glucosidase-inhibitory activities [11] of *P. neochilus* have been reported, but prevailing is the antimicrobial activity. In a recent paper, it was demonstrated that even after being used for phytoremediation, the essential oils from this plant conserves its high antimicrobial profile [12].

A few years ago, *P. neochilus* was introduced in Cuba and quickly started to be cultivated because of its ethnobotanical and pharmacological benefits and its ornamental properties [13]. Recently, an ethnobotanical survey revealed that this plant is mainly consumed by the Cuban population for its sedative and hypnotic effects rather than for its traditional antimicrobial and antidiabetic properties [14]. Due to the recent introduction, its wide use, and the lack of information other than ethnobotany on this species in Cuba, the purpose of this research was to investigate the pharmacognostic parameters, the phytochemical profile of leaves and extracts of *P. neochilus* cultivated in Cuba, as well as its antimicrobial activity.

## 2. Results

The apparent inconsistent ethnomedicinal use of *P. neochilus* extracts against sleeping disorders in Cuba and microbial infections elsewhere motivated us to explore potential differences in pharmacognostic, phytochemical, antimicrobial, and cytotoxic properties of Cuban cultivars. For this purpose, four lots of plant material (February, May, August, and November of 2018) were harvested.

### 2.1. Plant Quality Control Parameters

#### 2.1.1. Fresh Leaves

Macroscopic determinations did not reveal phenotypical differences throughout the year of study (four lots collected) and are in agreement with the deposited botanical information for this plant in Cuba [13] and Brazil [15]. Leaves with creased edges, perinervic venation, petiole with a wedge-shaped base, membranous texture, greenish-gray color, and a strong and characteristic aromatic odor are the most representative macroscopic quality control parameters. Other remarkable characteristics are an adaxial and abaxial pubescent leaf surface, with generally short hairs. In spite of the strong and characteristic aromatic odor, none of the eight determined essential oil (EO) yields (two replicates for the four lots) allows us to obtain quantifiable values greater than the sensitivity of the determination method. Accordingly, the EO yield values (EOY) measured did not exceed the detection limit (EOY < 0.01%). In general, the EO is detected at relatively low yields. For the species that grows in Brazil, values around 0.03% have been regularly reported [6,16], while for the species that grow in Portugal [8] and South Africa, yields exceed 0.2% [17].

**Figure 1:**
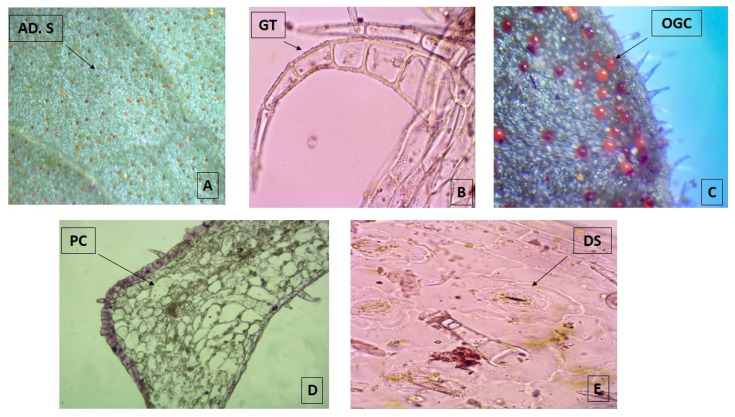
Photomicrograph of transverse sections of leaves from *Plectranthus neochilus* collected in Santiago de Cuba. (**A**) Adaxial surface of the foliar lamina. (**B**) Non-glandular trichomes. (**C**) Orange-colored glandular cells. (**D**) Polygonal cells. (**E**) Diacytic stomata. (**A**) × 40× and (**B**–**E**) × 100×.

The micro-morphological analysis revealed that both leaf surfaces are covered with trichomes and abundant orange-colored glandular cells that are more evident in the adaxial surface when observed under the stereo-microscope 40× (Figure 1A). The multicellular and uni-serial nature of non-glandular trichomes (Figure 1B), as well as the orange-colored glandular cells (Figure 1C), can be better observed when using higher magnifications of the bright field microscope at 100×. The adaxial surface has a cuticle of approximately 1 μm thick with a single layer epidermis with polygonal cells of 10 μm in size, periclinal and anticline walls, tracing straight or convex lines that are up to 1.25 µm thick (Figure 1D). The abaxial epidermis is also 1.25 µm thick without a cuticle and has diacytic stomata (Figure 1E) with a density of 22.5/100 µm^2^. The parenchyma is homogeneous, lacunar, with five to six strata of amorphous cells reaching a thickness of 35 µm. Most of these observations match with the micro-morphological characteristics described for the species, which were recently updated, with the only exception of the presence of glandular trichomes [18].

#### 2.1.2. Dry Leaves

The drying process was carried out by sun-drying, shade-drying, and oven-drying (45 °C). Due to the succulent characteristics, none of the air-exposure methods was effective since more than three weeks were needed to achieve drying, causing microbiological contamination in the case of shade-dried batches. Under these circumstances, only the oven-dried method was able to eliminate the water from the leaves in 6–8 days, preserving most of the organoleptic characteristics. The residual moisture content was 10.5 ± 1.7% (azeotropic method) (n = 8, two determinations on the four batches).

The other quality control parameters repeatable within batches (Table 1) showed no appreciable differences between the different harvesting moments. In all cases, it was possible to define an upper and lower decision limit, allowing to predict a range in which the results should be expected. Even when no statistical differences (*p* > 0.05) among batches were found for none of the quality control parameters, the batch selected to prepare the extracts from *P. neochilus* leaves was batch 2 (May), considering the maximal number of soluble compounds.

### 2.2. Plant Extracts and Quality Control Determinations

#### 2.2.1. Physical and Physicochemical Parameters of Plant and Extracts

Three extracts were prepared: the first following the ethnobotanical information [14] using water and fresh, crushed leaves (FLD). The other two extracts used dried leaves and water (DLW) or commercial ethanol at 94% (DLE) as solvents. All three extracts were prepared using the leaf material coming from batch 2. Table 2 summarizes the physical and physicochemical parameters of the extracts (three replicates).

As can be seen in Table 2, the quality control parameters are different in all extracts, especially those of a quantitative character. Water stands out as a solvent with a high capacity to extract constituents of *P. neochilus* leaves. In relation to pH, dried leaves favor the extraction of acidic compounds.

#### 2.2.2. Determination of the Phytochemical Profile of the Extracts

**Figure 2:**
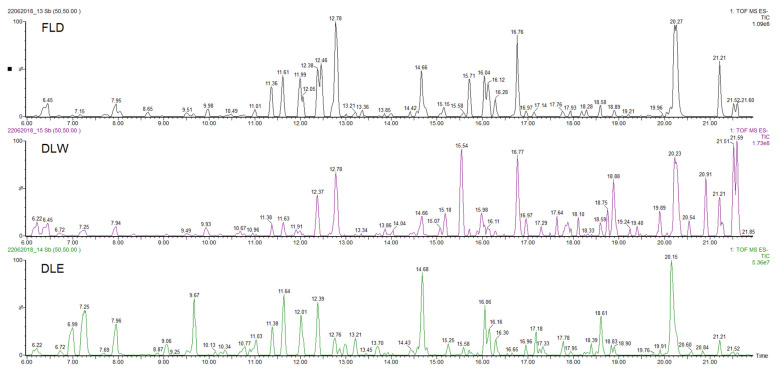
UPLC-DAD-MS/MS Total Ion Chromatogram (TIC) profiles of *Plectranthus neochilus* Schltr. extracts.

The three extracts of *P. neochilus* FLD, DLW, and DLE were analyzed by UPLC-DAD-MS/MS. Visual evaluation of the total ion chromatogram (TIC) of all extracts (Figure 2) revealed a resemblance between the extracts with regard to the position of peaks but displayed a difference in relation to the relative amount of the compounds present. This was confirmed by the dereplication analysis performed and in-depth exploration of the main peaks in the three chromatograms, which reveals that the major changes observed are related to changes in relative intensity of the peaks.

Dereplication is a strategy that provides fast identification of known metabolites in complex biological mixtures, speeding up the process to identify natural products [19], while Feature-Based Molecular Networking (FBMN), available on the Global Natural Products Social Molecular Networking (GNPS) web platform, supports the analysis of the LC-MS/MS data. This process rendered 22 library hits. The matched compounds were mainly glycosides, glucuronides, and methoxy derivates of quercetin, kaempferol, luteolin, and apigenin; abietane-type diterpenoids, fatty acyl derivates, and rosmarinic acid; helping the further process of compound identification.

Considering that the aim of this research is to evaluate why Cuban cultivars are not typically used for their potential antimicrobial effects (most reported worldwide activity), the chemical metabolite identification was focused on the extract obtained by the method that most closely resembles the inhabitants’ way of preparation (FLD). Peaks with at least 20% of relative intensity were considered, and their presence was evaluated in the other two studied extracts (DLW and DLE). Under these conditions, 18 peaks were selected and tentatively identified (Table 3, Figure 2).

Compound **1**, with a retention time of 6.45 min, shows a peak with *m*/*z* 387.1647 [M − H]^−^ yielding a fragment at *m*/*z* 207 [M-H-C_6_H_12_O_6_]-due to the loss of a neutral hexoside residue (180 Da) and another at *m*/*z* 163 [M-H-C_6_H_12_O_6_-COO-]- as result of the loss of the carboxyl function.

**Figure 3:**
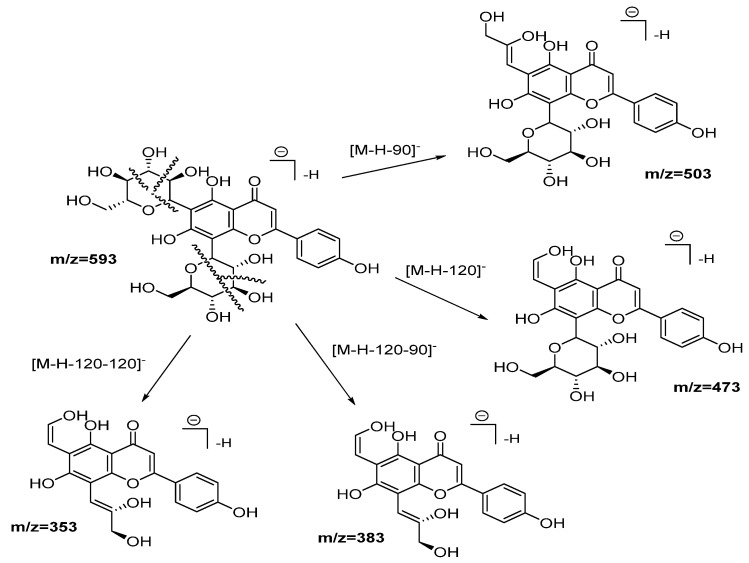
Fragmentation pathways (ESI negative mode) proposed for Vicenin 2.

Compounds from **2** to **5**, as well as **10**, **12**, **13**, and **14**, were identified as flavonoid (flavone and flavonol type) derivates in which the nature of the aglycone was inferred with the help of the MS2 ESI positive mode [20]. Compound **2** was identified as vicenin-2. Figure 3 shows the fragmentation pattern proposed assigning those product ions as a result of the cross-ring cleavages in di-hexose *C*-flavonoid glycoside [20,21]. The UV spectra confirm the flavonoid nature of compound **2** with 225, 270, and 375 nm peaks.

Compounds **3** and **5** were classified as position isomers, showing similar molecular ions and fragments consistent with glucuronide loss (176 Da). As a unique difference, peak 3 displays an extra fragment at *m*/*z* 476 with relatively high intensity (51%), suggesting an easier loss of a methyl group from an ether moiety than peak 5 that, on the contrary, shows the fragment *m*/*z* 299 with higher intensity. For compound **3**, a peak at 153.0118 (ESI positive mode) confirms that the –OCH_3_ moiety is not present in the A-ring. Those observations, together with the information derived from the FBMN analysis, and the chemotaxonomic information for the genus *Plectranthus* [22], allows us to assign position 4′ and 7 for the methoxy groups of compounds 3 and 5, respectively, while the glucuronide group was placed at position 3.

**Figure 4:**
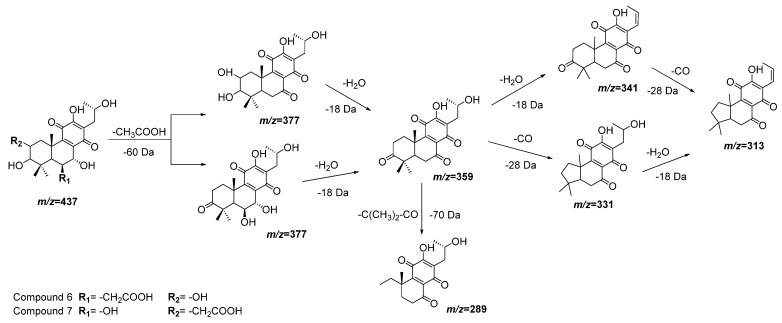
Fragmentation pathways (ESI negative mode) proposed for compounds **6** and **7** in *Plectranthus neochilus* extracts.

Compounds **6** and **7** also have almost the same molecular ions and similar initial fragmentation patterns representing the loss of an acetyl group (−60 Da) and a water molecule (−18 Da). The difference between both compounds is defined by the rest of the fragments and their relative intensity. Considering the results of dereplication analysis and the abundance of reports of abietane diterpenoids for *Plectranthus* species [23,24], Figure 4 is proposed as fragmentation pathways for compounds **6** and **7**. The position of the hydroxyl and acetyl substituents are conditioned to the cleavage of the ring A (-C(CH_3_)_2_-CO, *m*/*z* = −70 Da), which is only possible in compound **7** after the loss of the acetyl group of position 2.

Compound **8** was coincident with a fractionation pattern of a fatty acyl glycoside and tentatively identified as 2-(8-(hydroxymethoxy)oct-1-en-3-yloxy)-hexoside-pentose. Fragment ions at *m*/*z* 437 and 421 are in correspondence with the cleavage on both sides of the ether bond of the aliphatic chain. The base fragment (100% intensity) at *m*/*z* 289 is a consequence of the further loss of the pentoside unit.

Compound **9** and its fragmentation pattern allowed us to identify this peak as the well-known compound rosmarinic acid. Both of the fragment ions at *m*/*z* 197 and 179 correspond to the cleavage of the ester group, while fragment *m*/*z* 161 corresponds to the loss of two or one water molecules of each one of the previous fragments, respectively. The last fragment observed, *m*/*z* 135 (even in a very low intensity), confirms the assigned substance. Based on the peak area at UV detection, this compound classifies as the main compound in this extract. This result is in concordance with previous information [25].

**Figure 5:**
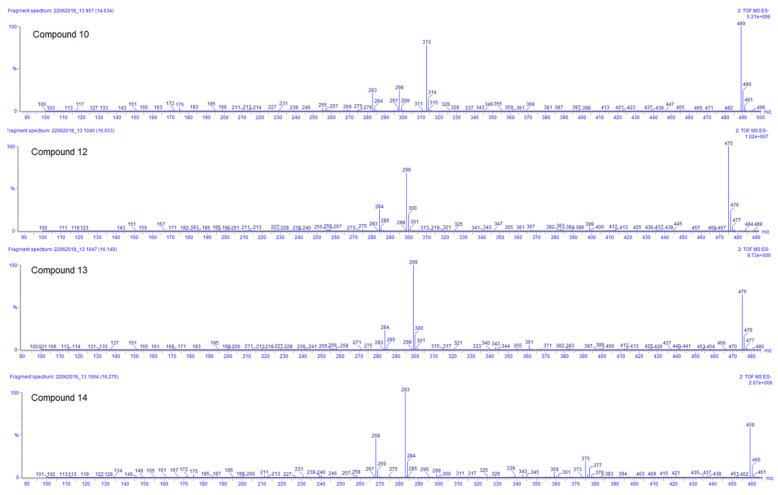
ESI negative ion mode MS^2^ spectra of compounds **10**, **12**, **13**, and **14** of *Plectranthus neochilus* extracts.

Compounds **10, 12, 13**, and **14** are the last flavonoids identified. All are methoxy-glucuronide-flavonoid derivates. Typical for all compounds is the loss of the glucuronyl group generating the fragments at *m*/*z* 313, 299, 299, and 283, respectively (Table 3). In the case of the first two, this fragment results in the second most abundant, while for compounds **13** and **14**, the fragments *m*/*z* 299 and 283, respectively, represent the main peak in the spectrum (see Figure 5). Further fragments are in agreement with the loss of methyl groups from the methyl ether (−15 Da).

For compound **10**, the ESI positive mode shows a distinctive fragment at 179 (0,4B+), characteristic of the C–C cleavage at positions 0/4 of luteolin [20]. Considering all those facts, the chemotaxonomic information available, and the relative intensity of the [M − H]^−^, it is suggested that the glucuronyl group is attached at position 7, while methoxy groups are placed at 3′ and 4′ [20,26]. The ESI positive mode of compound **12** shows fragments at *m*/*z* 121 (0,2B+) and 165 (0,2A+), characteristic of the C–C cleavage at positions 0/2 of kaempferol [20], the rest of the factors are similar to compound **10**. Compound **13** has almost the same MS and UV characteristics to compound **12**. The only remarkable difference between compounds **13** and **12** is related to the relative intensity of the mass peaks. For compound **13** the main fragment was found at *m*/*z* 299, resulting from the loss of the glucuronyl, compared to compound **12** where the pseudomolecular ion [M − H]^–^ was seen as the main ion in the spectrum (see Figure 5). This allowed us to infer that in flavonoid **13** the glucuronyl substituent is placed in the ‘non-favored’, position 3 [26]. At last, compound **14** corresponds to an apigenin derivate considering the fragment in ESI positive mode of *m*/*z* 163 (0,4B+). The main fragment (*m*/*z* 283) does not correspond to the pseudomolecular ion [M − H] ^–^; therefore, the glucuronyl substituent was linked to the ‘less favored’ apigenin hydroxyl substituent position 5 [26]. The methoxy group was arbitrarily placed in position 7 or 4′.

**Figure 6:**
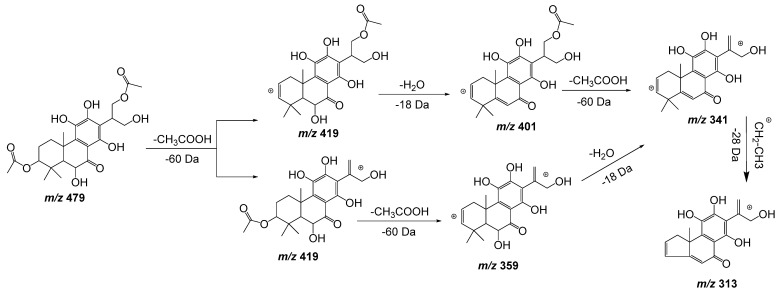
Fragmentation pathway (ESI negative mode) proposed for compound **11**: 6,11,12,14,18-pentahydroxy-3,17diacetyl-8,11,13-triene-7-one.

Compound **11**, with a pseudomolecular ion at *m*/*z* 479.1918 [M − H] ^–^ and molecular formula C_24_H_32_O_10_, was inferred as a diacetylditerpenoid. The consecutive loss of both acetyl fragments followed by the loss of a water molecule and the similarity with coleon D and coleon U compounds isolated from *P. barbatus*, *P. fasciculatus*, *P. forsteri*, *P. grandidentatus*, *P. madagascariensis*, *P. nummularius*, *P. sanguineus*, *P. argentatus*, and *P. myrianthus* [27], allows us to identify compound **11** as 6,11,12,14,18-pentahydroxy-3,17diacetyl-8,11,13-triene-7-one (Figure 6).

Compound **15** fragments suggest a water molecule (18 Da) *trans*-elimination followed by the loss of a carboxyl group (44 Da), respectively. The additional loss of 162 Da indicates the presence of a hexose unit. This fragmentation pattern, the FBMN analysis, and the chemotaxonomic information available for *Plectranthus* genus allow us to tentatively identify compound **15** as hexosyl-6β-hydroxicarnosol. The aglycone 6β-hydroxicarnosol has already been reported for *Plectranthus barbatus* [28]. The unfavored loss of the hexosyl group is associated to the steric hindrance provoked by 7,20-epoxyabieta-20-one moiety, being consistent with the β position.

Compound **16** seems to have a similar backbone as compounds **6** and **7** but with an additional double bond. The fragments suggest the same sequence of fractionation with the loss of H_2_O (−18 Da), CH_3_COOH (−60 Da), H_2_O (−18 Da), and at last CO (−28 Da). This information allows us to identify compound **16** as 3,6,11,12,14-pentahydroxy-2-acetyl- 5,7,11,13-abietatetraen-7-one. Similar compounds have been isolated from *P. madagascariensis* [27] and *P. scutellarioides* [24].

**Figure 7:**
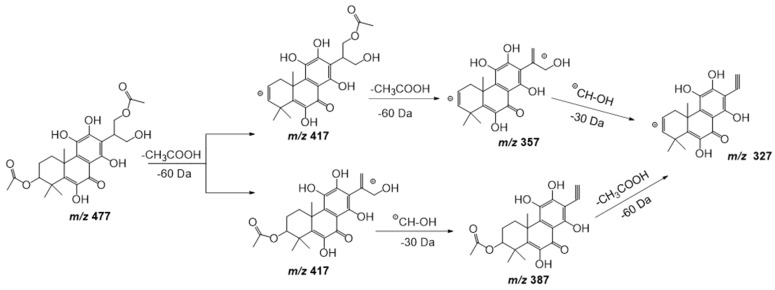
Fragmentation pathway (ESI negative mode) proposed for compound **17**: 6,11,12,14,16-pentahydroxy-3,17diacetyl-5,8,11,13-tetraene-7-one.

Compound **17** shows a pseudomolecular ion at *m*/*z* 477.1798 [M − H] ^–^ corresponding to the molecular formula C_24_H_30_O_10_, being similar to compound **11** but with an extra double bond. Figure 7 shows the fragmentation pathway proposed for this compound. This metabolite has been previously isolated from *P. scutellarioides* [24].

The last compound identified seems to have a similar abietane backbone as compounds **6** and **7**, as well as compound **17**. The fragment at *m*/*z* 359 (second most abundant with 51%) can be explained by the loss of a neutral acetyl group [M-H-60] ^–^ with the consequent oxidation of the adjacent hydroxyl group at position 3, as described before with compound **7** (see Figure 4). The fragment, with a very low relative intensity (6%) at *m*/*z* 341, represents the loss of a water molecule. Based on this information, the hydroxyl group should be positioned at place 6, generating a double bond. The other hydroxyl substituent was placed in a position that does not allow easy water loss, position 12 (as chemotaxonomic pattern in most of the abietane diterpenoids identified in *Plectranthus genre*). All this information was also supported by the FBMN analysis. The coleon U-quinone nucleus suggested for compound **18** has been recurrently reported for *Plectranthus* species, such as *P. madagascariensis*, *P. sanguineus*, *P. forsterii*, *P. grandidentatus*, and *P. myrianthus* [27].

### 2.3. Antimicrobial Activity

The antimicrobial activity was evaluated against a wide panel of microorganisms, consisting of two bacteria (*Staphylococcus aureus* and *Escherichia coli*), one yeast (*Candida albicans*), one fungus (*Aspergillus fumigatus*), and five parasites (*Leishmania infantum*, *Leishmania amazonensis*, *Trypanosoma cruzi*, *Trypanosoma brucei brucei*, and *Trypanosoma brucei rhodesiense*). Neither aqueous extract (FLD and DLW) showed any significant antimicrobial activity, in agreement with the Cuban ethnobotanic information [14], but in contrast to other international reports [6,9]. The ethanol extract of the dried leaves (DLE) is the only extract with good activity against *Trypanosoma* species (Table 4). None of the microorganisms was sensitive to any of the three extract preparations.

### 2.4. Cytotoxicity

Three different cell lines were used to test the influence of the extracts on cell viability: human fetal lung fibroblasts MRC-5SV2, murine macrophage RAW 264.7, and human monocytic THP-1cells to evaluate the safety and/or toxic action of *P. neochilus* extracts. The results showed that for the three cell lines, the most toxic extract (DLE) is also non-selective (Table 5). On the other hand, the aqueous extracts do not affect cell viability at the maximum concentration tested. Therefore, when combining this result with the moderate activity observed on some of the parasites, these extracts are classified as moderately selective (Table 5).

## 3. Discussion

From the macromorphological and phenotypic point of view, there is no difference between the plants that grow in Cuba and those reported in the bibliography. However, the essential oil production never reached an amount that would allow quantification in any of the four batches. Similarly, when analyzing micro-morphological aspects, most of the parameters of the plant under study match the international descriptions [18,29], but no glandular trichomes were found in either abaxial or adaxial leaf surfaces. At the same time, we did not find any remarkable antimicrobial activity, using a validated and well standardized primary screening microbiological strain panel, which suggests that Cuban cultivars lack antimicrobial properties and antiseptic use of extracts should not be recommended to Cuban inhabitants, as recommended for other cultivars [30,31]. This antimicrobial activity not only refers to human pathogens but also phytopathogens, as was recently reported [32].

Glandular trichomes play a fundamental role in the storage and excretion of volatile substances that attract or repel natural enemies. Their absence in the samples studied in the present work could be the explanation for such low production of essential oils, which probably remain stored in the internal vesicles maintaining the characteristic smell of the plant. It is internationally accepted that biotic and abiotic aspects induce changes, especially in sedentary organisms, such as plants. This becomes more evident in tropical islands, such as Cuba, in which the climatic change increases the aridity and salinity of the soils due to the combined effect of increased drought and high levels of temperature and evaporation [33]. In a recent study, a GC-MS-based metabolomics approach was used to explain the effect of environmental factors on the volatile composition of *P. neochilus*. Seasonal air temperature and rainfall were some of the variables considered. On the other hand, Milaneze-Gutierre et al. highlighted the fact that a variation in the quantity and color of these trichomes is possible depending on climatic conditions and stored metabolites [34]. This phenomenon is known as plant plasticity [35].

*Plectranthus neochilus* is a plant native to South Africa (a completely different ecosystem from Cuba), but nevertheless, it is distributed worldwide; quantitative and qualitative chemical differences are recognized with respect to EO production. While in the coldest regions of South Africa and Portugal, the yield in the EO production reaches at least 0.2%, and the main constituent is α-terpenyl acetate, in the plantations of Brazil, citronellol is the main constituent and yields barely reach 0.03%. The decrease or even the cancellation of the EO synthesis by drought or high temperature has been described before in the literature [4]. Under this premise, it is possible to hypothesize that in Cuban climatic conditions (high temperatures and evaporation levels), *P. neochilus* ‘protects’ its EO production in internal vesicles instead of in glandular trichomes exposed to the sun, making it less accessible for extraction.

Another consequence described by the effects of drought or high temperatures is the reduction of photosynthesis. This results in an increment of the production of phenolic and/or polyhydroxylated compounds as a way to counteract the increased production of reactive oxygen species [36]. In the present study, 16 of the 18 identified compounds show a high oxidation pattern. In general, isolated compounds were classified into two main metabolites types: flavonoids and abietane diterpenoids. From the eight flavonoids identified, seven have at least six oxygen atoms in their structure, and five shows a free or methoxylated catechol moiety in ring B, indicative of a high oxidation pattern. Reports of flavonoids in the *Plectranthus* genus are not abundant, but most of them are flavones and flavonols [22]. Exactly these types of flavonoids were identified in this study: flavones (compounds **2**, **4**, **10**, and **14**) and flavonols (compounds **3**, **5**, **12**, and **13**). Recently, the presence of rutin was reported for *P. neochilus* [30] and even when this compound was not isolated in this work, compounds **3** and **5** are structurally close. On the other side, abietane diterpenoids are commonly reported in the *Plectranthus* genus [27]. In this paper, we tentatively identified seven abietane diterpenoids (compounds **6**, **7**, **11**, **15**, **16**, **17**, and **18**), and with the exception of compound **15**, in the other six, the oxidation degree can be considered as high (with a mean of eight to ten oxygen atoms within the diterpene backbone). Due to the scarce information available of the non-volatile *P. neochilus* metabolites, to the best of our knowledge, all the metabolites identified, with the exception of rosmarinic acid (compound **9**), are reported for the first time in this species. Moreover, high levels of phenolic and/or polyhydroxylated compounds may further contribute to the divergent phytochemical composition and properties of Cuban cultivars of *P. neochilus* in these particular climatic conditions.

Relative to the cytotoxic profile, aqueous extracts (FLD and DLW) result in low toxicity to the different panel cells, while the ethanol extract (DLE) is classified as cytotoxic and not selective. This behavior is in agreement with the recent report of Matias and collaborators that classified three different water extracts as non-cytotoxic but a non-polar extract as moderately cytotoxic [30]. Ethanol tends to extract non-polar metabolites, such as diterpenes. Several extracted diterpenes, especially in the genus *Plectranthus*, can act as cytotoxic agents [37,38].

Overall, our results showing moderate antimicrobial activity of Cuban cultivars of *P. neochilus* are in full agreement with the Cuban ethnobotanical information, in contrast to reports on *P. neochilus* species grown elsewhere. Most of the information available from the species under study is related to essential oil production and its association with antimicrobial activity [6,8,9,16,39]. In particular, the 2014 article by Mota et al. [8] demonstrated significant antimicrobial activity on two bacterial strains, *S. aureus* and *E. coli*, included in our study. Presumably, the low levels of EO produced by the *P. neochilus* cultivars in Cuba may reduce its antimicrobial efficacy and, as such, may contribute to the lack of popularity of *P. neochilus* extracts for antiseptic use by Cuban inhabitants.

Altogether, and to the best of our knowledge, this study reveals for the first time, the pharmacognostic, phytochemical, and biological profile of Cuban cultivars of *P. neochilus*.

## 4. Materials and Methods

### 4.1. Plant Collection

Adult plants were collected in the morning from the experimentation plot of the National Centre of Applied Electromagnetism (Lat 20.032316, Lon −75.810585) at the Universidad de Oriente, Santiago de Cuba. Plant authentication was carried out in the Eastern Center for Biodiversity and Ecosystems (BIOECO) in Santiago de Cuba with a voucher specimen (code JSC.147) deposited at Jorge Sierra Calzado Herbarium of the aforementioned center.

### 4.2. Quality Control Drug Parameters Determinations

Four lots of plant material (February, May, August, and November of 2018) were harvested. The collected leaves were divided into two parts: Fresh leaves were used for macroscopic, microscopic, and EO determinations, while the remaining part was dried using three different drying methods: sun-dried, shade-dried, and oven-dried (45 °C). The drying process of the leaves was considered effective when the following four criteria were accomplished: (1) at least the three last weight determinations did not show a coefficient of variation of more than 0.1%; (2) the drying period did not exceed three weeks (21 days); (3) no evident microbial contamination appeared; and (4) the residual moisture determined by the infrared method (Sartorius MA 35, Göttingen, Germany) was equal or less than 14% [40].

#### 4.2.1. Macroscopic and EOY Determinations

Fresh leaves from the four lots were analyzed in detail for their anatomic characteristics, size, shape, smell, and color by a stereo-microscope NOVEL NSZ-606 (Nanjing, China) with 40× magnification. In addition, an EO extraction process by hydro-distillation (3 h) was performed using 200 g of fresh leaves in a Clevenger apparatus for the four lot samples. Results were expressed in percentage of EOY (mean value of the two determinations) as previously described [41].

#### 4.2.2. Microscopic Determination

For the histological studies, four leaves were selected from each collected lot. Cross-leaf sections were dehydrated in alcoholic series of 50%, 70%, 75%, and 90%, and stained with a mixture of safranin and 90% alcohol. After staining the cross-sections, they were mounted in glycerinated gelatin and sealed with paraffin. Sections 8-μm thick were obtained using a microtome Kedee202 A (Zhejiang, China). The sections were died with safranin and methylene blue, cleared in xylol, and ridded in Canadian Balsam as previously described [42]. The epidermis, cell wall, stomata, and mesophyll were characterized according to the methodology described by Theobald and collaborators [43]. The cuts were observed and photographed in a microscope Novel N-220M (Nanjing, China) coupled with a Canon digital camera 1600 × 1200 pixels at 400×.

#### 4.2.3. Total Ash Content and Total Extractable Matter

These parameters were determined following the protocols suggested by the WHO [44]. Accordingly, we calculated the mean value of the three replicates for each of the four lots.

For the determination of the total ash content, 2 g of the dry plant material was incinerated for 2 h in an incandescent silicate crucible at 700 °C (Boeco MF 8/1100 muffler, Hamburg, Germany). This process was repeated until constant mass. The cooled residue was weighed, expressing the values in percent.

Total soluble substances in water and ethanol were determined using the cold maceration method. Five grams of the dry plant material were placed in a 250-mL Erlenmeyer flask with a frosted top lid, and 100 mL of the solvent (water or ethanol) was added. After 12 h of stirring maceration and 6 h repose, the content was filtered, and 25 mL of the filtrate was transferred to flat-bottomed dishes to evaporate the solvents at 105 °C in a Carl Roth SC 150 thermostatted bath (Karlsruhe, Germany). The cooled residue was weighed, expressing the values (*w/v*) in percent.

### 4.3. Plant Extracts Preparation and Quality Control Determinations

Three different kinds of extracts were prepared: one following the indications derived from the ethnobotanical information [14] consisting of a water decoction of 10 g of the fresh, crushed leaves in 100 mL of water (FLD). The other two extracts were obtained in the same proportion but using dried leaves, which were subjected to maceration for 48 h with either water (DLW) or commercial ethanol at 94% (DLE) as solvents.

#### 4.3.1. Determination of the Physical and Physicochemical Parameters of the Extracts

The determination of organoleptic characteristics, pH value, and total extractable substances were performed following standard protocols [45]. Organoleptic properties were evaluated by simple inspection of their appearance (color, texture, and smell). The pH value was measured with a calibrated pH meter (Hanna Instruments, Eibar, Spain). Total extractable substances were determined by a gravimetric method after drying 5 mL of the extracts in a porcelain capsule. The results of these last two parameters are reported as the mean and standard deviation (SD) of three determinations.

#### 4.3.2. Determination of the Phytochemical Profile of the Extracts

Chemical composition was analyzed in a UPLC-DAD-MS/MS system using a Xevo G2-XS QTof spectrometer (Waters, Milford, MA, USA) coupled with an ACQUITY LC system equipped with MassLynx version 4.1 software. Five µL of each extract (FLD, DLW, DLE) at 100 µg/mL were injected onto a BEH Shield RP18 column (100 mm × 2.10 mm, 1.7 µm; Waters, Milford, MA, USA). The mobile phase solvents consisted of H_2_O + 0.1% FA (A) and ACN + 0.1% FA(B), and the gradient was set as follows (min/B%): 0.0/10, 5.0/10, 20.0/15, 30/15, 40.0/25, 45.0/25, 55.0/40, 60.0/40, 65.0/100, 70.0/100, 75.0/10, 85.0/10.

Full scan data were recorded in ESI (−) and ESI (+) modes from *m*/*z* 50 to 1500, and the analyzer was set to sensitivity mode (approximate resolution: 22,000 FWHM). The spray voltage was set at either +1.5 or −1.0 kV; cone gas flow and desolvation gas flow at 50.0 and 1000.0 L/h, respectively; and source temperature and desolvation temperature at 120 and 550 °C, respectively. Data were also recorded using MSE in the positive and negative ionization modes (two analyses per mode), and a ramp collision energy from 20 to 30 V was applied to obtain additional structural information. Leucine Encephalin was used as lock mass. DAD spectra were recorded between 190 and 500 nm.

Using the software ReifycsAbf Converter, the UPLC-DAD-MS/MS raw data were converted to abf files and processed with MS-DIAL version 4.24 [46] for mass signal extraction between 50 and 1500 Da from 0 to 22 min (range in which the main peaks appears). A centroid mode with a tolerance of 0.01 and an optimized detection threshold of 8000 for MS1 and 5000 for MS2 was fixed. The Global Natural Products Social (GNPS, https://gnps.ucsd.edu) (accessed on 3 January 2022) export function of MS-DIAL [47] was used to export the results and created a molecular network with the Feature-Based Molecular Networking (FBMN) workflow [48]. Major peaks were tentatively characterized by means of MS/MS spectra comparing with those found in the literature and the public databases PubChem (https://pubchem.ncbi.nlm.nih.gov/) (accessed on 3 January 2022), ChemSpider (https://www.chemspider.com/) (accessed on 3 January 2022), MassBank of North America (MoNA) (http://mona.fiehnlab.ucdavis.edu/) (accessed on 3 January 2022), and NIST Mass Spectrometry Data Center (http://chemdata.nist.gov/) (accessed on 3 January 2022).

### 4.4. Determination of the Antimicrobial Activity

The antimicrobial activity was classified as good (IC_50_ ≤ 20 μg/mL), moderate (20 μg/mL < IC_50_ ≤ 64 μg/mL), or inactive (IC_50_ > 64 μg/mL) according to the criteria of Cos and collaborators [49].

#### 4.4.1. Microorganisms

The antimicrobial screening was performed against nine microorganisms, including two bacteria (*S. aureus* ATCC 6538, *E. coli* ATCC8739), one yeast (*C. albicans* ATCC B59630 (azole-resistant)), one fungus (*A. fumigatus* ATCC16404), and five parasites (*L. infantum* MHOM/MA (BE)/67), *L. amazonensis* (MHOM/BR/77/LTB0016), *T. cruzi* (Tulahuen CL2), β-galactosidase, *T. brucei brucei* (Squib 427), and *T. brucei rhodesiense* (STIB-900)). All strains were supplied by the culture collection of the Laboratory for Microbiology, Parasitology and Hygiene (LMPH), University of Antwerp, Belgium, except for *L. amazonensis*, which was supplied by the Institute of Tropical Medicine Pedro Kourí, Havana, Cuba.

#### 4.4.2. Strain Culture and Extract Dilution

Mueller Hinton Broth (MHB) was used for bacteria culture and maintained on TSA (Tryptone Soy Agar), while RPMI-1640 medium supplemented with MOPS buffer and glucose was used for fungi culture and maintained on PDA (Potato Dextrose Agar). *Leishmania infantum* parasites were maintained in the Golden Hamster (Mesocricetus auratus); while *L. amazonensis* were routinely isolated from an infected BALB/c mouse and directly cultivated in Schneider’s medium (Sigma-Aldrich, St. Louis, MO, USA) containing 10% heat-inactivated fetal bovine serum (HFBS, Sigma-Aldrich) and antibiotics (100 μg of streptomycin/mL and 100 U of penicillin/mL). Hirumi (HMI-9) medium, supplemented with 10% inactivated fetal calf serum was used to maintain *T. brucei brucei* and *T. brucei rhodesiense*; while *T. cruzi* was cultured in human lung fibroblast (MRC-5SV2) cells in Minimum Essential Medium Eagle (MEM) supplemented with 200 mM L-glutamine, 16.5 mM NaHCO3, and 5% inactivated fetal calf serum.

To obtain extract concentrations in which the solvent DMSO does not exceed 1% concentration, powdered extracts (lyophilized) were re-dissolved in DMSO to create a stock solution (equivalent to 20 mg/mL). It was subsequently serially diluted four-fold in de-mineralized water to a concentration range from 0.25 to 128 μg/mL.

#### 4.4.3. Antibacterial and Antifungal Assays

Test compounds were diluted in sterile 96-well microplates with the redox-sensitive dye resazurin [49]. For this purpose, 10 μL of the test extract concentration was added to each well together with 190 μL of bacteria inoculum (5 × 10^5^ CFU/mL) or yeast inoculum (5 × 10^3^ CFU/mL). Incubation time varied depending on the microorganism from 7 days at 27 °C (mold), 24 h at 37 °C (yeast), and 17 h at 37 °C (bacteria). After this time, 10 μL of resazurin at 50 μg/mL per well was added. After this incubation period (under the same temperature condition previously declared) and times ranging from two and one days for, respectively, *A. fumigatus* and *C. albicans*, and 15 and 45 min for, respectively, *S. aureus* and *E. coli*. Corresponding fluorescence signals were measured at λ_ex_ = 550 nm and λ_em_ = 590 nm in a microplate reader (TECAN GENios, Germany). Microbial growth after treatment was compared to untreated-control wells (100% cell growth) and medium-control wells (0% cell growth). Doxycycline, flucytosine, and terbinafine (all purchased from Sigma-Aldrich, St. Louis, MO, USA) were used as a reference drugs. Mean IC_50_ values estimated using linear regression analysis are presented with the SD of replicates (n = 2).

#### 4.4.4. Antileishmanial Assays

Antileishmanial assay: Amastigotes of both *Leishmania* strains were pelleted from the spleen of the infected donor hosts using RPMI medium (SIGMA, St. Louis, MO, USA) following three centrifugation steps. The spleen parasite burdens were assessed using the Stauber technique (Stauber, 1966). Primary peritoneal mouse macrophages (PMM) were used as host cells, collected 2 days after peritoneal stimulation with a 2% potato starch suspension. Isolated PMM were incubated for 2 h at 37 °C and 5% CO_2_ removing the non-adherent cells. Amastigotes of *L. infantum* or stationary-phase promastigotes of *L. amazonensis* were added at a proportion of 10:1 or 4:1 parasite/macrophage adherent cells and incubated for 24 or 4 h, respectively, at 37 °C and 5% CO_2_ in RPMI medium supplemented with heat-inactivated fetal bovine serum. After the free parasites were discarded, an additional five and two incubation days at the same conditions for *L. infantum* and *L. amazonensis*, respectively, was performed. Parasite burdens were microscopically assessed after staining the cells with a 10% Giemsa solution, and these parasite burdens (mean number of amastigotes/macrophage) were used to evaluate the activity. The results are expressed as the percent reduction in parasite burden compared to untreated control wells, calculating the IC_50_ from linear regression curves. Two replicates of all experiments were carried expressing the medium of IC_50_ and the standard deviation. Glucantime^®^ (Rhône-Poulenc Rorer, Mexico) and miltefosine (Sigma-Aldrich, St. Louis, MO, USA) were used as reference drugs for *L. amazonensis* and *L. infantum*, respectively.

#### 4.4.5. Antitrypanosomal Assays

The assays were performed in 96-microwell plates containing 190 μL of the parasite suspension of trypomastigotes (at least 1.5 × 10^4^ parasite/well for *T. brucei brucei* and 4 × 10^3^ parasite/well for *T. brucei rhodesiense*, respectively) and 10 μL of the extract dilution to be tested. The plates were incubated for three days, and then 50 μL resazurin was added per well to be incubated again for 6 h (*T. rhodesiense*) or 24 h (*T. brucei*) at 37 °C. Parasite growth was determined by measuring the fluorescence intensities (λ_ex_ 550, λ_em_ 590 nm) in a microplate reader. Untreated-infected wells were calibrated as 100% parasite growth, while uninfected medium controls were defined as 0% growth. Suramin (Sigma-Aldrich, St. Louis, MO, USA) was used as a reference drug while the influence of the extract on the parasite growth/viability was expressed in percent.

For *T. cruzi* evaluation, 190 μL of the cell/parasite inoculum was seeded in sterile 96-well microtiter plates, consisting of a mixture of 4 × 10^3^ cells/well of MRC-5SV2 (human fetal lung fibroblasts) and 4 × 10^4^ parasites/well with 10 μL of the extract dilution to be tested. The parasite growth was compared with untreated infected controls (100% growth) and non-infected controls (0% growth) after seven days of incubation. The parasite burden was assessed after adding chlorophenol red-β-D-galactopyranoside (CPRG): 50 μL/well of a stock solution containing 15.2 mg of CPRG and 250 μL Nonidet in 100 mL phosphate buffered saline (PBS). Then, the change in color was measured at 540 nm after 4 h of incubation at 37 °C. The results are expressed as a percent reduction in parasite burdens compared to control wells, and an IC_50_ was calculated. Benznidazole (Sigma-Aldrich, St. Louis, MO, USA) was used as a reference drug. Two replicates of all experiments for the three *Trypanosoma* strains were performed.

#### 4.4.6. Cytotoxicity Assays

Cellular cytotoxicity of the different extracts was evaluated in human fetal lung fibroblasts MRC-5SV2 cells, murine macrophage (RAW 264.7), and human monocytic (THP-1) cell lines (ATCC, American Type Culture Collection). MRC-5SV2 cells were cultured in MEM plus Earl’s salts-medium, supplemented with L-glutamine (20 mM), 16.5 mM sodium hydrogen carbonate, and 5% inactivated fetal calf serum (FCS). RAW 264.7 and THP-1 cell lines were grown in RPMI 1640 + 10% FCS + 2mM L-glutamine.

Cells were seeded in 190 μL (1.5 × 10^5^ cells/mL) in DMEM without FCS, 2% L-glutamine, and glucose/conc. 4.5 g/L (D-glucose) in sterile 96-well microtiter plates. Different doses of extracts were added in 10 μL medium volume (final concentration 256, 128, 64, 32, 16, and 8 μg/mL) and incubated at 37 °C and 5% CO_2_ for 72 h. After this incubation time, 50 μL/well resazurin solution (2.2 μg/mL) was added and after 4 h incubation under the same conditions; the cell viability was assessed fluorometrically in a microplate reader (TECAN GENios, Crailsheim, Germany) at λ_ex_ 550 nm, λ_em_ 590 nm. Untreated-control wells were calibrated as 100% cell growth, while medium-control wells were set at 0% cell growth. The results were expressed as a percent reduction of cell growth/viability compared to control wells, and corresponding IC_50_ values were determined. Tamoxifen (Sigma-Aldrich, MO, USA) was used as a reference drug. Upon dividing both IC_50_ values, the Selectivity Index (SI) was calculated for each microorganism. Different extract samples were classified in selective (SI ≥ 5), moderately selective (3 ≤ SI < 5), and unspecific or non-selective (SI < 3).

### 4.5. Statistical Analysis

Statistical analysis was carried out in GraphPad Prism 8 (GraphPad Software, San Diego, CA, USA). Tukey’s test was used as a multiple comparison test for independent samples to define significant differences (*p*-value under 0.05) between the quantitative quality control parameters of dried leaf lots and derived physico-chemical parameters of extracts. Before statistical data analysis, a normal distribution was verified using the Kolmogorov–Smirnov test implemented in the same software. Decision limits for upper and lower cut-off values of the standard deviations were estimated, when possible, by each quantitative quality control parameter for a 95% confidence.

## 5. Conclusions

In the present investigation, the pharmacognostic, phytochemical, and biologic profiles of Cuban cultivars of *P. neochilus* were analyzed for the first time. Macro and micro-morphological analyses revealed a low yield of essential oil production and the absence of glandular trichomes compared to plants growing at other latitudes. Different quality control parameters were evaluated for fresh and dried leaves, as well as for the prepared extracts. Traditional leaf extraction by Cuban inhabitants was compared to the extraction of dried leaves by aqueous or ethanol solvents. The three extracts mainly vary in the ratio in which the main components are present, but not too much in their chemical composition. A moderate antimicrobial activity, as well as low cytotoxicity, was detected for the aqueous extracts (FLD and DLW) compared with the ethanol extract (DLE), which showed a significant antimicrobial activity but also high cytotoxicity. The low yield of essential oil production, the absence of glandular trichomes, and the high number of hydroxyl substituents in most of the compounds identified were considered as critical determinants of the relatively moderate antimicrobial effects observed for the extracts of the Cuban cultivars grown in particular climate conditions, compared to cultivars grown at other latitudes. Alternatively, the potential sedative properties of Cuban cultivars of *P. neochilus* to improve sleep quality need to be further investigated.

## Figures and Tables

**Table 1:** Quantitative quality control parameters of dried leaves from *Plectranthus neochilus* collected in Santiago de Cuba at different times.

Parameter	Batch 1 (February)	Batch 2 (May)	Batch 3 (August)	Batch 4 (November)	LDL (95%)	UDL (95%)
Total ash content (%)	8.1 ^a^ ± 1.4	8.4 ^a^ ± 2.0	9.7 ^a^ ± 1.3	8.5 ^a^ ± 1.7	4.8	12.5
Ethanol total soluble substances (%)	18.5 ^b^ ± 2.0	20.2 ^b^ ± 2.7	17.5 ^b^ ± 1.9	20.2 ^b^ ± 0.4	14.5	23.7
Water total soluble substances (%)	22.6 ^c^ ± 1.3	24.1 ^c^ ± 1.4	22.1 ^c^ ± 1.1	23.5 ^c^ ± 1.6	19.8	26.3

LDL; Lower Decision limit, UDL; Upper Decision limit. Equal letters within the same row indicate no statistical differences (LSD Tukey test, α = 0.05).

**Table 2:** Physical and physicochemical parameters of the extracts prepared from batch 2 (May 2018) of Plectranthus neochilus leaves grown in Santiago de Cuba.

Parameter	Fresh Leaves Decoction (FLD)	Dry Leaves Water Maceration (DLW)	Dry Leaves Ethanol Maceration (DLE)
Organoleptic characteristics	Color: light green Smell: characteristic of the plant Texture: slightly dense	Color: light brown Smell: characteristic of the plant Texture: slightly turbid	Color: dark green Smell: characteristic of the plant Texture: transparent
Total extractable substances (%)	15.99 ^b^ ± 0.01	20.19 ^c^ ± 0.01	10.67 ^a^ ± 0.01
pH	5.27 ^c^ ± 0.01	4.85 ^b^ ± 0.02	4.12 ^a^ ± 0.01

Different letters within the same row indicate statistical differences (LSD Tukey test, α = 0.05).

**Table 3:** Assigned compounds, [M − H]^−^ and ESI negative fragment ions of the eighteen peaks detected in *Plectranthus neochilus* fresh leaves decoction (FLD) extract.

Compound	Rt (min)	Accurate Mass [M − H]^−^ (*m/z*)	Error (ppm)	MS/MS Ions (Rel. Intensity, %)	Molecular Formula	Tentative Identification	AEP
**1**	6.45	387.1647	−1.3	207(17), 163(8)	C_18_H_28_O_9_	12-Hydroxyjasmonic acid glucoside	DLW
**2**	7.95	593.1553	1.5	503(12), 473(37), 413(5), 383(11), 353(19)	C_27_H_30_O_15_	Vicenin-2	DLW, DLE
**3**	11.36	491.0858	−1.0	475(51), 315(59), 299(64)	C_22_H_20_O_13_	4′-Methoxy-quercetin-3-*O*-glucuronide	DLW, DLE
**4**	11.61	461.0721	−0.9	285(57), 255(22)	C_21_H_18_O_12_	Luteolin-*O*-glucuronide	DLW, DLE
**5**	11.99	491.0829	0.4	315(69), 299(33)	C_22_H_20_O_13_	7-Methoxy-quercetin-3-*O*-glucuronide	DLW, DLE *
**6**	12.05	437.1805	0.7	377(100), 359(86), 341(22), 331(30), 315(62)	C_22_H_30_O_9_	3,6,7,12,16-Pentahydroxy-2-acetyl-5,8,12-abietatrien-11,14-dione	DLW *, DLE *
**7**	12.38	437.1816	−0.2	377(38), 359(41), 289(71)	C_22_H_30_O_9_	2,3,7,12,16-Pentahydroxy-6-acetyl-5,8,12-abietatrien-11,14-dion	DLW, DLE
**8**	12.46	467.2131	0.4	437(18), 421(36), 289(100)	C_20_H_36_O_12_	2-(8-(Hydroxymethoxy)oct-1-en-3-yloxy)-hexoside-pentose	None
**9**	12.79	359.0778	1.6	197(25), 179(23), 161(48), 135(7)	C_18_H_15_O_8_	Rosmarinic acid	DLW, DLE
**10**	14.66	489.1032	−1.4	313(57), 298(19), 283(18)	C_23_H_22_O_12_	3′,4′-Dimethoxy-luteolin-7-glucuronide	DLW, DLE
**11**	15.71	479.1918	−1.5	419(86), 401(62), 359(41), 341(24), 313(21)	C_24_H_32_O_10_	6,11,12,14,16-Pentahydroxy-3,17diacetyl-8,11,13-abietatrien-7-one	DLW *, DLE *
**12**	16.04	475.0871	−1.3	299(73), 284(31)	C_22_H_20_O_12_	Methoxy-kaempferol-7-glucuronide	DLW *, DLE
**13**	16.12	475.0874	−0.6	299(100), 284(39)	C_22_H_20_O_12_	Methoxy-kaempferol-3-glucuronide	DLW *
**14**	16.28	459.0930	−0.4	283(100), 268(51)	C_22_H_20_O_11_	Methoxy-apigenin-5-glucuronide	DLW, DLE
**15**	16.76	511.2578	1.0	493(27), 467(76), 305(9)	C_26_H_40_O_10_	Hexosyl-6β-hydroxicarnosol	DLW
**16**	18.58	435.1661	1.1	375(42), 357(19),327(9)	C_22_H_28_O_9_	3,6,11,12,14-Pentahydroxy-2-acetyl-5,7,11,13-abietatetraen-7-one	DLW, DLE
**17**	20.27	477.1798	−1.0	417(100), 387(17), 357(23), 327(11)	C_24_H_30_O_10_	6,11,12,14,16-Pentahydroxy-3,17-diacetyl-5,8,11,13-abietatetraen-7-one	DLW
**18**	21.21	419.1721	−1.4	359(51), 341(6)	C_22_H_28_O_8_	3,6,12-Trihydroxy-2-acetyl-8,12-abietadien-7,11,14-trione	DLW, DLE

Rt→Retention Time, AEP→Peak presence in alternative extract (DLW and DLE), *→Traces.

**Table 4:** Antimicrobial activity of extracts from *Plectranthus neochilus* leaves growing in Santiago de Cuba.

Extract	IC_50_ ± SD (μg/mL)								
	*S. aureus*	*E. coli*	*C. albicans*	*A. fumigatus*	*T. cruzi*	*T. b. brucei*	*T. b. rhodesiense*	*L. infantum*	*L. amazonensis*
FLD	>128	>128	70.7 ± 2.4	>128	>128	>128	64.4 ± 1.2	>128	>128
DLW	>128	>128	>128	>128	>128	>128	>128	60.1 ± 3.2	>128
DLE	56.6 ± 1.8	>128	>128	>128	16.6 ± 0.6	16.6 ± 1.0	16.1 ± 0.5	64.9 ± 2.1	>128
Doxycycline	0.04 ± 0.0	0.6 ± 0.0	-	-	-	-	-	-	-
Flucytosine	-	-	0.7 ± 0.0	-	-	-	-	-	-
Terbinafine	-	-	-	0.3 ± 0.0	-	-	-	-	-
Benznidazol	-	-	-	-	1.8 ± 0.0	-	-	-	-
Suramine	-	-	-	-	-	0.02 ± 0.0	0.03 ± 0.0	-	-
Miltefosine	-	-	-	-	-	-	-	10.8 ± 1.3	
Glucantime	-	-	-	-	-	-	-	-	12.3 ± 2.9

**Table 5:** Cytotoxicity and Selectivity index (SI) of extracts from *Plectranthus neochilus* leaves growing in Santiago de Cuba.

Extract	IC_50_ ± SD (µg/mL)			SI
	MRC-5	RAW 264.7	THP-1	
FLD	>256	>256	>256	≈4 (*C. albicans and T. brucei rhodesiense)*
DLW	>256	>256	>256	≈4 (*L. infantum)*
DLE	<16	<16	<32	≈1
Tamoxifen	8.3 ± 1.1	10.9 ± 2.3	10.3 ± 2.1	-

## Data Availability

All data are available in the article.

## Abbreviations

ATCC	American Type Culture Collection
AEP	Peak presence in alternative extract
BIOECO	Center for Biodiversity and Ecosystems
CPRG	Chlorophenol red-β-D-galactopyranoside
DMSO	Dimethyl sulfoxide
DLW	Dry leaves water maceration
DLE	Dry leaves ethanol maceration
EO	Essential oils
EOY	Essential oil yield
ESI	Electro Spray Ionization
FBMN	Feature-Based Molecular Networking
FCS	Fetal calf serum
FLD	Fresh leaves decoction
GNPS	Global Natural Products Social Molecular Networking
HFBS	Heat-inactivated fetal bovine serum
IC_50_	Inhibitory concentration
LMPH	Laboratory for Microbiology, Parasitology, and Hygiene
MEM	Minimum Essential Medium Eagle
MHB	Mueller Hinton Broth
MoNA	MassBank of North America
NIST	National Institute of Standards and Technology
PDA	(Potato Dextrose Agar)
PBS	Phosphate buffered saline
PMM	Peritoneal macrophages from mice
Rt	Retention Time
SD	Standard deviation
SI	Selectivity Index
TSA	Tryptone Soy Agar
TIC	Total Ion Chromatogram
UPLC-DAD-MS/MS	Ultra-high performance liquid chromatography-Diode Array Detection-Mass/Mass WHO: World Health Organization
